# Cytokine Storm in COVID-19: Immunopathogenesis and Therapy

**DOI:** 10.3390/medicina58020144

**Published:** 2022-01-18

**Authors:** Christian Zanza, Tatsiana Romenskaya, Alice Chiara Manetti, Francesco Franceschi, Raffaele La Russa, Giuseppe Bertozzi, Aniello Maiese, Gabriele Savioli, Gianpietro Volonnino, Yaroslava Longhitano

**Affiliations:** 1 Foundation “Ospedale Alba-Bra Onlus”, 12060 Verduno, Italy; christian.zanza@live.it (C.Z.); lon.yaro@gmail.com (Y.L.); 2 Department of Emergency Medicine, Anesthesia and Critical Care Medicine, Michele and Pietro Ferrero Hospital, 12060 Verduno, Italy; 3 Department of Emergency Medicine, Policlinico Gemelli/IRCCS University of Catholic of Sacred Heart, 00168 Rome, Italy; francesco.franceschi@unicatt.it; 4 Research Training Innovation Infrastructure, Research and Innovation Department, Azienda Ospedaliera SS Antonio e Biagio e Cesare Arrigo, 15121 Alessandria, Italy; 5 Department of Anesthesia and Critical Care Medicine, St. Antonio and Biagio and Cesare Arrigo Hospital, 15121 Alessandria, Italy; Tatsiana_romenskaya@yahoo.it; 6 Department of Surgical, Medical, and Molecular Pathology and Critical Care Medicine, Institute of Legal Medicine, University of Pisa, via Roma 55, 56126 Pisa, Italy; a.manetti3@studenti.unipi.it (A.C.M.); aniello.maiese@unipi.it (A.M.); 7 Department of Clinical and Experimental Medicine, University of Foggia, 71122 Foggia, Italy; gius.brt@gmail.com; 8 Emergency Medicine and Surgery, IRCCS Fondazione Policlinico San Matteo, 27100 Pavia, Italy; gabrielesavioli@gmail.com; 9 Department of Anatomical, Histological, Forensic and Orthopaedic Sciences, Sapienza University of Rome, Viale Regina Elena 336, 00161 Rome, Italy; gianpietro.volonnino@uniroma1.it

**Keywords:** cytokine storm, COVID-19, glucocorticoids, anakinra, tocilizumab, JAK inhibitors, methylene blue, plasmapheresis

## Abstract

A cytokine storm is a hyperinflammatory state secondary to the excessive production of cytokines by a deregulated immune system. It manifests clinically as an influenza-like syndrome, which can be complicated by multi-organ failure and coagulopathy, leading, in the most severe cases, even to death. The term cytokine storm was first used in 1993 to describe the graft-versus-host disease following allogeneic hematopoietic stem cell transplantation. It was then reused to define the adverse syndromes secondary to the administration of immunostimulating agents, such as anti-CD28 antibodies or bioengineered immune cells, i.e., CAR T-cell therapy. Currently, the concept of cytokine storm has been better elucidated and extended to the pathogenesis of many other conditions, such as sepsis, autoinflammatory disease, primary and secondary hemophagocytic lymphohistiocytosis, and multicentric Castleman disease. Moreover, cytokine storm has recently emerged as a key aspect in the novel Coronavirus disease 2019, as affected patients show high levels of several key pro-inflammatory cytokines, such as IL-1, IL-2, IL-6, TNF-α, IFN-γ, IP-10, GM-CSF, MCP-1, and IL-10, some of which also correlate with disease severity. Therefore, since the onset of the pandemic, numerous agents have been tested in the effort to mitigate the cytokine storm in COVID-19 patients, some of which are effective in reducing mortality, especially in critically ill patients, and are now becoming standards of care, such as glucocorticoids or some cytokine inhibitors. However, the challenge is still far from being met, and other therapeutic strategies are being tested in the hope that we can eventually overcome the disease.

## 1. Introduction

Cytokines are polypeptides that act as intercellular mediators. They are essential for the correct functioning of the immune system and are involved in a multiplicity of pathophysiological processes fundamental for survival, such as inflammation, tissue repair, fibrosis, and coagulation. However, when produced in excess due to a dysfunction of the immune system, cytokines may become harmful to the body, leading to a state of systemic hyperinflammation called a cytokine storm [1]. Cytokine storm underlies several pathological conditions, such as autoinflammatory diseases, primary and secondary hemophagocytic lymphohistiocytosis (HLH), multicentric Castleman disease, and sepsis, and has recently emerged as a key aspect in the novel Coronavirus disease 2019 (COVID-19). For this reason, many researchers have tried to implement therapies to mitigate the cytokine storm in the treatment of COVID-19 patients, with various results [2]. In this article, we summarize the current knowledge on the etiopathogenesis of cytokine storms, analyzing its role in COVID-19 and providing a critical analysis of the therapies used to treat it.

## 2. Etiopathogenesis of the Cytokine Storm

From a teleological perspective, the task of the immune system is to recognize the presence of a harmful agent within the body, giving an inflammatory response to promote its elimination, supporting damage repair, and finally returning to the basal state. Everything is orchestrated by cytokines, a real language through which immune cells communicate and coordinate with each other. A complex network of regulatory mechanisms guarantees the balance between the production of pro-inflammatory and anti-inflammatory cytokines so that the reaction remains limited, commensurate with the pathogenic noxa, and is exhausted when the latter is eliminated. The failure of one or more of these mechanisms can lead to immune system overactivation and massive production of cytokines, causing an inflammatory reaction that is no longer localized but systemic, with harmful consequences for the whole organism [3].

Whether on a genetic or acquired basis, dysfunction can reside at any level, and the production of cytokines can occur in the absence of any trigger, as in autoinflammatory diseases and idiopathic Castleman disease, or following the recognition of a non-pathogenic trigger, as in the case of macrophage activation syndrome (MAS), a subset of secondary hemophagocytic lymphohistiocytosis (sHLH) caused by autoimmune diseases. An infectious agent can overwhelm the body’s defenses, leading to systemic involvement, as in sepsis, or escape the clearance of the immune system, causing its prolonged stimulation, as in Epstein-Barr virus-related HLH. On the other hand, the immune system may defect in efficiently eliminating the antigenic stimulus to return to homeostasis, as in the case of primary HLH. Finally, immune hyperactivation can occur due to iatrogenic causes, following the administration of drugs that stimulate the immune system (such as the anti- CD-19 anti-CD3 monoclonal antibody Blinatumomab) or bioengineered cells (chimeric antigen receptor T-cell therapy) [4,5,6,7,8]. In Table 1, the clinical conditions associated with the cytokine storm are shown.

Innate and adaptative immunity cells are involved in the genesis of the cytokine storm, as well as macrophages, for example, in the following conditions: MAS, CAR T-cell therapy-associated cytokine release syndrome, severe acute respiratory syndrome (SARS), and Middle East respiratory syndrome (MERS) [3,8,9,10,11,12]. Activated macrophages can produce several pro-inflammatory cytokines, such as tumor necrosis factor (TNF) α, interleukin (IL)-1, IL-6, and IL-18, which may trigger the inflammatory cascade, generating a cytokine storm. Hemophagocytic macrophages are often observed in the bone marrow and other tissues of patients affected by cytokine storms, maybe contributing to the cytopenia commonly observed in this condition [13].

Natural killer (NK) cells are the main cells responsible for cytokine storm in primary HLH, in which a defect in their cytolytic function leads to prolonged antigenic stimulation and incapability of resolving inflammation [6,14,15]. Similarly, NK cells’ function can be impaired in the presence of IL-6 excess, which lowers perforin and granzyme production and hence may contribute to other forms of the cytokine storm. Neutrophils can produce neutrophil extracellular traps, a network of fibers that contributes to thrombi formation and amplifies cytokine production during the storm [13]. Endothelial cells may also be involved: following their activation by mediators such as IL-6 and IFN-γ, in turn, these cells start to produce further pro-inflammatory cytokines and can contribute to the onset of coagulopathy [16,17].

The ability of T lymphocytes to initiate a cytokine storm is well exemplified by the effects of the iatrogenic hyperactivation of these cells in the context of CAR T cell and anti-CD28 antibody therapy [7,8]. Several T lymphocyte subtypes may be involved in different scenarios. Type 1 helper T cells (Th1) can produce large quantities of interferon (IFN) γ, activate macrophages, and are responsible for delayed hypersensitivity reactions [18]. T helper 17 cells (Th17) recruit neutrophils and can lead to autoimmunity reactions. Moreover, they can initiate a cytokine storm in experimental models of MAS [19,20]. Cytotoxic T cells (CTLs) work to eliminate infected or cancer cells with prolonged activation of the immune system; they also participate in the pathogenesis of sHLH [9]. Although the role of B lymphocytes in the genesis of the cytokine storm is considered secondary, the efficacy of therapies targeting these cells in conditions such as MCD suggests that they may also contribute in particular cases [21].

## 3. Clinical Features of the Cytokine Storm 

Although manifestations may vary based on the underlying pathology, a cytokine storm typically manifests as an influenza-like syndrome that may evolve or be complicated by multi-organ damage [13,22,23,24]. Fever is almost constant, with very high body temperature in the most severe cases. Other common manifestations include fatigue, headache, arthromyalgia, diarrhea, lymphadenopathy, hepatosplenomegaly, sensory changes, and skin rash. Tachypnea and hypoxemia are often present and can evolve to acute respiratory distress syndrome (ARDS). Acute kidney injury, liver damage, and stress-related cardiomyopathy can also develop in severe patients. Capillary leak syndrome with anasarca and neurological involvement with encephalopathy are also some possible complications of the cytokine storm. Coagulation is impaired, with the possible occurrence of thrombotic phenomena evolving in disseminated intravasal coagulopathy; bleeding risk is also increased at the same time. Laboratory findings in cytokine storm include leukocytosis, leucopenia, anemia, thrombocytopenia, and elevated levels of C-reactive protein (CRP), ferritin, triglycerides, and D-dimer.

## 4. COVID-19 Related Cytokine Storm 

Severe acute respiratory syndrome coronavirus 2 (SARS-CoV-2) is a member of Coronaviridae, a family of enveloped, positive-sense, single-stranded ribonucleic acid (RNA) viruses which infect humans and other mammals [25]. It is transmitted by air and primarily affects the respiratory system. Previous studies demonstrated that severe acute respiratory syndrome coronavirus 1 (SARS-CoV-1) and Middle East respiratory syndrome coronavirus (MERS) were both capable of inducing a cytokine storm [26]. Hence, after the advent of SARS-CoV-2, cytokine storm was advocated as a key pathogenetic factor in COVID-19. Numerous studies have shown that COVID-19 patients have increased levels of numerous inflammatory cytokines, including IL-1β, IL-2, IL-6, IL-10, IFN-γ, TNF-α, IFN-γ-inducible protein 10 (IP-10), granulocyte macrophage-colony stimulating factor (GM-CSF), and monocyte chemoattractant protein-1 (MCP-1), and that these cytokines correlate with the disease severity [27,28,29,30,31]. Many studies also demonstrated the presence of inflammatory infiltrates within various tissues in COVID-19 patients, both from bioptic and autoptic samples [32,33,34]. In most cases, the disease consists of a self-limiting flu-like syndrome; however, in predisposed subjects, the infection of lung cells, in particular of type II pneumocytes, can cause the recall of a rich inflammatory cell infiltrate, consisting of neutrophils, macrophages, CD8+ and CD4+ T lymphocytes, and massive production of cytokines, leading to bilateral pneumonia, ARDS, and multi-organ damage [35]. 

The inadequate immune response to the virus has been proposed as a possible mechanism in the development of cytokine storms during SARS-CoV-2 infection [36,37]. As demonstrated by Blanco-Melo and colleagues, infected cells show an impaired capacity to produce interferons, key mediators for a properly host response against viral infections; at the same time, they produce high levels of neutrophil- and macrophage-recruitant chemokines [38]. Additionally, different research groups have demonstrated that COVID-19 patients produce autoantibodies against several immuno-modulatory proteins and, in particular, the presence of anti-type I interferon antibodies is associated with severe disease and death [39,40]. Therefore, in the first phase of infection, the innate immune system may not be able to efficiently clear the infected cells and, on the contrary, could favor the replication of the virus. Consistently with this hypothesis, Jiadi Lv et al. observed that SARS-CoV-2 survives and replicates inside macrophages [41]. In a second phase, the immune system would recover the ability to effectively fight the virus, but since the latter has been able to replicate undisturbed, it would at that point produce an exaggerated reaction.

Some authors have also suggested a role of the renin-angiotensin-aldosterone system (RAAS) [35,42,43]. In fact, the main receptor used by SARS-CoV-2 to enter human cells is angiotensin-converting enzyme 2 (ACE2), a transmembrane glycoprotein with enzymatic activity belonging to RAAS. It is known that the local RAAS can mediate pro-inflammatory, prothrombotic, and profibrotic effects, mediated by the activation of the type 1 angiotensin II (AT1) receptor by angiotensin II. However, these effects are normally counterbalanced by the ACE2 receptor, which cleaves angiotensin I and II, generating a series of peptides with RAAS-antagonistic effects. It is believed that SARS-CoV-2, after binding the ACE2 receptor, causes its internalization in the cell and/or cleavage by cellular proteases such as disintegrin and metalloproteinase with thrombospondin motif 17 (ADAMT17), causing tissue downregulation of ACE2. Given these premises, it has been hypothesized that the loss of ACE2-mediated anti-inflammatory, antithrombotic, and anti-fibrotic effects, as well as the simultaneous relative upregulation of the angiotensin II-AT1 axis, may contribute to the genesis of the cytokine storm and the thrombo-inflammatory state characteristic of COVID-19.

## 5. Targeting the Cytokine Storm in COVID-19

### 5.1. Anti-Inflammatory/Immunosuppressive Agents

Chloroquine and hydroxychloroquine were widely used at the beginning of the pandemic. They seemed to be promising drugs for the treatment of COVID-19 due to their multiple pharmacological properties. They inhibit the synthesis of cytokines, such as IL-1 and IL-6, but they also enhance the production of other inflammatory mediators [44]. Therefore, chloroquine and hydroxychloroquine have anti-inflammatory effects. Moreover, they showed antiviral activity against a broad spectrum of microorganisms, including SARS-CoV-1. Preliminary in vitro and in vivo findings seemed to confirm their beneficial effect against SAR-CoV-2 infection [45,46]. Nevertheless, data from randomized controlled trials (RCTs) and subsequent metanalysis demonstrated that they have little or no impact on COVID-19 prevention or treatment. Furthermore, they were associated with an increased risk of adverse events [47]. 

Well-known for their numerous immunosuppressive and anti-inflammatory properties, glucocorticoids are used to treat a great number of inflammatory and autoimmune conditions. They can act at various levels on the genesis and maintenance of the cytokine storm. For this reason, glucocorticoids were empirically prescribed to COVID-19 patients. They then became part of standard therapy following the publication of the Randomised Evaluation of COVID-19 Therapy (RECOVERY) trial results, which demonstrated a significant reduction in mortality with the administration of dexamethasone in hospitalized patients on oxygen therapy or mechanical ventilation [48]. Several metanalyses have confirmed these results [49,50,51]. 

Colchicine is an anti-inflammatory drug used to treat gout, pericarditis, and familial Mediterranean fever, which acts by inhibiting activation and migration of neutrophils and by interfering with the inflammasome complex, essential for IL-1β and IL-18 production [52]. An RCT showed the potential benefit in the treatment of SARS-CoV-2 infection, but further research did not confirm these data [53,54,55]. Therefore, colchicine is not recommended by current guidelines.

### 5.2. RAAS Targeting Drugs

**Figure 1:**
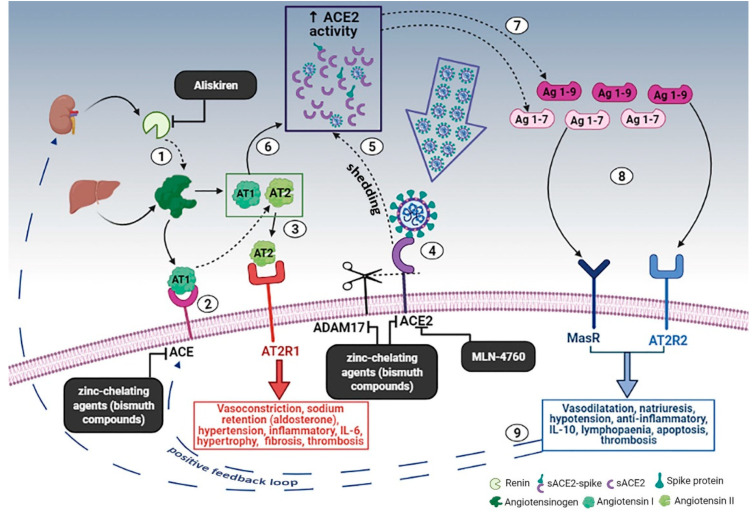
Schematic diagram of the effects of the RAAS system during SARS-CoV-2 infection and the proposed treatment. (1) Renin secreted by the kidney cleaves angiotensinogen, produced by the liver, to form AT1; (2) AT1 is converted to AT2 by pulmonary ACE. (3) AT2 binds to AT2R1 (angiotensin II receptors 1). The excess of AT2 through AT2R1 hyperactivation causes vasoconstriction, sodium retention (by aldosterone release), hypertension, inflammatory, IL-6, hypertrophy, fibrosis, and thrombosis. (4) SARS-CoV-2 binds to ACE2 to enter the host cell; however, the cellular protective response leads to ACE2 shedding. (5) ADAM17-regulated ectodomain shedding of ACE2 results in an increased amount of soluble and active ACE2 (sACE2). (6) AT1 and AT2 can also bind to sACE2. (7) They are then metabolized by ACE2 into Ag 1–9 and Ag 1–7, respectively. (8) The excess of Ag 1–9 and Ag 1–7 signaling via AT2R2 and MasR can induce vasodilatation, natriuresis, hypotension, anti-inflammatory effects, IL-10, lymphopenia, apoptosis, and thrombosis. (9) These events, in turn, produce a compensatory upregulation of both renin secretion and ACE activity, which establish the onset of a positive feedback loop. In the black boxes, drugs that can potentially stop the positive feedback loop by inhibiting enzymes of the RAAS are indicated. Dashed arrows indicate enzymatic activity, full arrows indicate non-enzymatic passage, and dashed blue arrows represent the positive feedback loop. Created in Bio-render.com. Reprinted from Ref. [42].

Based on the hypothesis that the RAAS can contribute to the genesis of the COVID-19-related cytokine storm, ACE inhibitors and sartans have been studied in patients with COVID-19, but their efficacy is still uncertain. While several retrospective studies have shown a potential protective effect, an RCT found no clinical benefit from the use of these drugs in patients with COVID-19 [56,57,58,59,60,61]. Further randomized clinical trials are currently underway and could help clarify the issue (NCT04335786, NCT04311177, NCT04328012). Another possible way to target the RAAS, although not yet tested, could be the use of a direct renin inhibitor, such as the Food and Drug Administration (FDA)-approved aliskiren, to reduce the production of AT1 (Figure 1).

### 5.3. Cytokine Inhibitors

With the emergence of biotechnological pharmacology, numerous cytokine inhibitors have been developed and successfully used in immune-rheumatological pathologies, including conditions characterized by cytokine storm syndrome (CSS). Hence, as the cytokine storm could also play a crucial role in the pathogenesis of COVID-19, researchers experimented with the use of several of these drugs in patients infected by SARS-CoV-2. IL-1 is produced following the activation of the innate immune system; it is one of the most important pro-inflammatory molecules involved in the cytokine storm. Anakinra is a recombinant form of the IL-1 receptor antagonist, which, similarly to its endogenous analog, is able to competitively inhibit the binding of IL-1β to its receptor IL1R. This drug has been used successfully in numerous autoimmune and auto-inflammatory conditions, including cytokine release syndrome (CRS) secondary to CAR-T cell therapy and HLH [62,63,64]. These characteristics have led to the experimentation of anakinra in COVID-19 patients with conflicting results. A series of cohort studies recorded a clear benefit from the use of this drug in severe forms of COVID-19 [65,66,67]. On the other hand, a recent RCT conducted in France was suspended prematurely for futility, as no advantage was appreciated in the treatment group [68]. However, the results of the latter study were contested because the sample was composed of patients with mild to moderate forms of the disease, characterized by relatively low levels of C reactive protein, thus excluding the patients who could most benefit from the inhibition of IL-1 (i.e., severe forms characterized by signs and symptoms of CSS). More data are needed to address the issue, and further RCTs are ongoing (NCT04357366, NCT02735707, NCT04339712). 

Another very promising target is represented by IL-6, given its important role in the pathogenesis of many forms of cytokine storms and the availability of specific inhibitors. Among these, particular interest was aroused by tocilizumab, an anti-IL-6 monoclonal antibody used in the treatment of rheumatoid arthritis, juvenile idiopathic systemic arthritis, cytokine storm secondary to CAR-T cell therapy, and Castleman disease [69,70,71,72]. The effects of tocilizumab in COVID-19 patients have been investigated by several research groups with mixed fates. In the CORIMUNO-TOCI2 trial, conducted in France on 131 COVID-19 patients with moderate-to-severe pneumonia, the authors were unable to demonstrate any mortality benefit in patients treated with tocilizumab [73]. Similarly, in the COVACTA trial, including 438 patients with COVID-19 pneumonia, no benefit on survival was noticed [74]. However, more recently, the results of two other trials reopened the game. The REMAP-CAP trial experimented with the use of tocilizumab and another IL-6 inhibitor (sarilimumab) in critically ill COVID-19 patients: patients treated with both drugs showed better outcomes, including survival, compared to standard care [75]. Similarly, in the RECOVERY trial, tocilizumab was shown to be effective in patients with hypoxia and hyperinflammatory state, improving various clinical outcomes, including 28-day mortality [76]. It should be noted that in both CORIMUNO and COVACTA trials, only a minority of patients were treated with corticosteroids. On the contrary, in the REMAP-CAP and in the RECOVERY trial, the use of glucocorticoids was more widespread and homogeneous in the different groups. These differences, given the proven effectiveness of glucocorticoids in COVID-19 patients and the possible existence of synergistic effects with tocilizumab co-administration, could explain such conflicting results. This hypothesis is further supported by the results of a recent meta-analysis on the effects of IL-6 inhibitors in COVID-19 [77]. The authors confirmed a reduction in mortality in patients treated with these drugs; however, this benefit seems to disappear in patients who had not also received cortisone drugs. Currently, the use of tocilizumab has been authorized for the treatment of selected patients in many countries. 

Given its multiple pro-inflammatory properties, TNF-α also has a strong rationale as a therapeutic target in COVID-19. Data extracted from the COVID-19 Global Rheumatology Alliance physician registry have shown a significant association between anti-TNF-α therapy and hospitalization length [78]; on these bases, at least two RCTs are further testing the efficacy of the anti-TNF antibodies adalimumab and infliximab (ISRCTN33260034, ISRCTN40580903). 

Rather than targeting a single molecule, Janus kinase (JAK) inhibitors can down-modulate the signaling pathways of multiple cytokines simultaneously: in fact, these agents act by inhibiting JAK, essential components of the Janus kinase-signal transducers and activators of the transcription (JAK-STAT) transduction system which mediate the cellular effects of several cytokine receptors, including IL-2, IL-6, IL-10, IFN-γ, and GM-CSF. JAK inhibitors have aroused particular interest following the positive results of two multicenter, double-blind, placebo-controlled trials. First, the Adaptive COVID-19 Treatment Trial 2 (ACTT-2) trial showed that the combination of baricitinib (a JAK inhibitor) plus remdesivir was associated with faster recovery compared to remdesivir alone [79]. Then, the Superiority Trial of Protease inhibition in COVID-19 (STOP-COVID19) trial found that administration of another JAK inhibitor, tofacitinib, in hospitalized patients with COVID-19 pneumonia was superior to standard care in a composite endpoint of respiratory failure and death [80]. However, it must be noticed that, in both trials, mortality was a secondary outcome, even if it favored treatment for baricitinib, as well as for tofacitinib; also of note, the STOP-COVID19 trial was funded by Pfizer, which produces tofacitinib. Nevertheless, these agents are now being tested in several other trials (e.g., NCT04421027, NCT04640168).

### 5.4. Antioxidants

The role of free radicals and oxidative stress in the pathogenesis of COVID-19 has been relatively denied. However, it is known that the recruitment and activation of immune cells in the context of numerous respiratory infections leads to the production of reactive oxygen and nitrogen species. These molecules contribute to the escalation of the inflammatory reaction, stimulating the synthesis of pro-inflammatory cytokines and damaging cell membranes through oxidative mechanisms, thus contributing to the onset of endothelial damage and thrombotic phenomena. In this sense, antioxidant agents could represent an intriguing adjunctive therapy in severe COVID-19 patients. Among others, particularly favorable properties are possessed by methylene blue, a tricyclic phenothiazine, approved as a therapy for methemoglobinemia and malaria, but also tested with success in other pathological conditions, including septic shock [81]. In the hyperinflammatory context of the cytokine storm, methylene blue may be able to simultaneously inhibit the production of reactive oxygen species (ROS), reactive nitrogen oxide species (RNOS), and cytokines themselves, thanks to its ability to counteract the generation of superanions by the xanthine oxidase pathway, to inhibit the synthesis of nitrous oxide (NO) by NO synthase, and to attenuate the nuclear factor-kB (NF-kB) signaling. In addition, in an in vitro study, methylene blue was shown to inhibit cellular infection by SARS-CoV-2 [82]. Currently, methylene blue has only been clinically tested in COVID-19 patients in a small, open-label, randomized trial in Iran. In this study, the authors observed reduced mortality in severely ill patients treated with methylene blue, although this represented a secondary outcome [83]. Another phase 2 clinical trial is ongoing in Switzerland (NCT04635605).

### 5.5. Blood Purification Therapies

An alternative, non-pharmacological approach to CSS is represented by blood purification therapies. Previous studies demonstrated that these techniques could remove cytokines from patients’ blood, and therefore, these techniques have been used in the treatment of various immune diseases [84]. In particular, therapeutic plasma exchange (TPE) was applied with promising results in several cytokine-storm-related conditions, such as HLH and sepsis [85,86,87,88]. Clinical results from many case reports, case series, and retrospective studies support TPE as an effective therapy in selected COVID-19 patients [89,90,91,92,93,94], but data from more structured RCTs are still lacking.

In Table 2, a summary of the main drugs and treatments which may be used during the cytokine storm in COVID-19 is provided.

### 5.6. Vaccine and Cytokine Storm

It has been suggested that COVID-19 vaccines may help prevent the cytokine storm through the early control of SARS-CoV-2 infection [95]. However, precise data concerning the prevalence of cytokine storms among vaccinated COVID-19 patients are not available. In the elderly, the “inflammaging” phenomenon may enhance cytokine release in COVID-19, but it may also reduce the immune response to the vaccine, as happens with other vaccines [96,97]. Therefore, the efficacy of COVID-19 vaccines in preventing the cytokine storm may be decreased in older patients [98]. To date, specific research concerning the actual effects of COVID-19 vaccines on the development of the cytokine storm has not been carried out or published. In vaccine clinical trials, researchers usually use disease severity, mortality, infection, transmission, or other surrogate endpoints to evaluate the vaccine efficacy, and the cytokine storm incidence is not studied [99]. Supposedly, the vaccine-induced immune response to the virus should prevent the cytokine storm by reducing the initial inflammatory reaction to the infection. Vaccine efficacy trials demonstrated high percentages of protection against severe disease [100]. However, new virus variants may decrease the vaccine’s effectiveness [100]. Moreover, a case of hyperacute reversible encephalopathy occurring the day after the administration of an adenoviral vector COVID-19 vaccine has been reported. In this paper, the authors suggested that the vaccine may have induced a cytokine storm which was causative of the encephalopathy [101]. Other authors also expressed concerns about the enhanced immune response after COVID-19 vaccines [102]. Further studies are needed to deepen the interplay between the vaccines and inflammatory response and to verify if the vaccination program is an effective strategy to prevent the cytokine storm. Furthermore, an important part of the population lacks access to vaccines, especially in developing countries, and the virus has a high mutation rate, which leads to the development of viral variants. Consequently, the vaccination program may be less effective, and the development of adequate therapies to treat the cytokine storm is still fundamental.

## 6. Conclusions

Recently, the development and large-scale delivery of vaccines effective in preventing SARS-CoV-2 infection raise the hope that the pandemic can finally be controlled. However, currently, a significant part of the population lacks access to vaccines, especially in developing countries; combining this with the high mutation rate (and the consequent development of viral variants) leads to the vaccines being less effective. All of this means that the development of effective therapies in the treatment of COVID-19 still represents a primary goal for public health. A better understanding of the pathogenesis of COVID-19 led to the implementation of therapeutic approaches that aim to counterbalance the immune system and cytokine storm with positive results, especially in the most severe forms of the disease. Many agents are still under study and further investigations will help to understand which approaches are the most effective, if and how they can be combined, and which patients could benefit from the newest therapy.

## Figures and Tables

**Table 1:** Clinical conditions associated with cytokine storm. Abbreviations: HLH, hemophagocytic lymphohistiocytosis; MCD, multicentric Castleman disease; HHV-8, human herpesvirus 8; EBV, Epstein-Barr virus; SARS, severe acute respiratory syndrome; MERS, Middle East respiratory syndrome; COVID-19, coronavirus disease 2019; CAR, chimeric antigen receptor.

Genetic/Idiopathic Diseases
Primary HLH Autoinflammatory diseases Idiopathic MCD
**Secondary HLH-associated diseases**
Autoimmune diseases Malignancies Intracellular pathogens
**Infective diseases**
Sepsis HHV-8 infection (secondary MCD) EBV infection Coronaviruses (SARS, MERS, COVID-19)
**Iatrogenic causes**
Blinatumomab CAR T cell therapy TGN1412

**Table 2:** A summary of the main drugs and treatments which may be used in the course of the cytokine storm in COVID-19. Abbreviations and symbols: ACEIs, angiotensin-converting enzyme inhibitors; MABs, monoclonal antibodies; MV, mechanical ventilation; TPE, therapeutic plasma exchange ↓, decreased; +, plus.

Drugs and Treatments	Mechanism of Action	Confirmed Benefit in Cytokine Storm?	References
Glucocorticoids	Immunosuppression and anti-inflammatory effects	↓ mortality (patients on O_2_ therapy or MV)	[48,49,50,51]
Colchicine	Neutrophils’ activation and migration inhibitor + interferes with the inflammasome	No benefit demonstrated	[53,54,55]
ACEIs	Angiotensin-converting enzyme inhibitor	Still uncertain	[56,57,58,59,60,61]
Sartans	Angiotensin receptor antagonist		
Aliskiren	Renin inhibitor		
Anakinra	IL1R antagonist	Still uncertain (seem to be effective)	[65,66,67,68]
Anti-IL6 MABs (tocilizumab)	IL6 inhibitors	Still uncertain (seem to be effective in addition to cortisone)	[73,74,75,76,77]
Anti-TNF MABs (adalimumab, infliximab)	TNF inhibitors	Still uncertain (further RCTs are ongoing)	[78]
Anti-JAK MABs (baricitinib, tofacitinib)	JAK inhibitors	Still uncertain (seem to be effective, further RCTs are ongoing)	[79,80]
Antioxidant agents (methylene blue)	ROS, RNOS, and cytokines production inhibitors (+ inhibit SARS-CoV-2 cellular infection?)	Still uncertain (further RCTs are ongoing)	[83]
Blood purification therapies (TPE)	Cytokines’ removal	Still uncertain	[89,90,91,92,93,94]
